# Psychometric Validity of the CES‐D Scale for Assessing Depressive Symptoms in University Students in Bogotá. Colombia

**DOI:** 10.1002/mpr.70072

**Published:** 2026-04-26

**Authors:** Angela Gissette Caro‐Delgado, Víctor Rivera Mancilla, Jorge Medina‐Parra, Freddy H. Villalobos‐Galvis

**Affiliations:** ^1^ Department of Psychology National University of Colombia Bogotá Colombia; ^2^ Delegation for the Right to Health and Social Security Defensoria del Pueblo Chapinero Colombia; ^3^ Vicepresidencia de innovación y Desarrollo Científico Clínica Reina Sofía Pediátrica y Mujer Clínica Infantil Santa María del Lago Clínicas Colsanitas Grupo Keralty Clínica Universitaria Colombia Bogotá Colombia; ^4^ Department of Pharmacy Faculty of Sciences Universidad Nacional de Colombia Bogotá Colombia; ^5^ Department of Psychology Psychology and Health Group University of Nariño Pasto Colombia

**Keywords:** cross‐sectional studies, depression, mental health, psychological tests, psychometrics, students

## Abstract

**Introduction:**

The Center for Epidemiological Studies Depression Scale (CES‐D) is a widely used tool for assessing depressive symptoms. This study examined its psychometric properties in a sample of 1738 university students in Bogotá.

**Methods:**

Instrumental study with a cross‐sectional, single‐center design with two‐stage sampling. Exploratory and two‐factor factorial analyses were applied, along with reliability estimates and subgroup analyses by gender and social stratum.

**Results:**

Exploratory factor analysis revealed a three‐factor structure (depressive affect, positive affect, and social aspects), which explained 56% of the total variance. The KMO index was 0.95, and Bartlett's sphericity test was significant (*χ* ^2^ = 17,902.82, df = 190, *p* < 0.001). The bifactor model showed a strong overall factor (hierarchical omega = 0.85; ECV = 0.71), supporting the use of a total score. Internal consistency was high (*α* = 0.93; *ω* = 0.94). Women scored significantly higher than men (*p* < 0.001). Differentiated cut‐off points by sex are proposed: ≥ 52 for women and ≥ 45 for men.

**Conclusions:**

These findings support the validity and reliability of the CES‐D as a unidimensional measure of depressive symptoms in the Colombian university population and highlight its usefulness as a screening tool in public health settings, particularly within Latin American regional contexts where early detection of mental health problems is a priority.

## 1 Introduction

Depression is one of the most prevalent mental disorders globally and constitutes a significant public health problem. The mental health of university students is a growing concern, with studies showing a high prevalence of disorders such as anxiety, depression (Chen and Lucock 2022; Duffy et al. 2020), and suicidal thoughts or behaviors (Mortier et al. 2018) in this population. This issue has been the subject of attention in the literature in recent years, with a significant increase in research since 2010 (Hernández‐Torrano et al. 2020). In the Region of the Americas, depression is among the top five causes of disease burden, and its control has been incorporated as a priority goal in the Sustainable Health Agenda 2018–2030, which urges Member States to “promote mental health and well‐being” as a cross‐cutting theme in public policies (Pan American Health Organization 2018).

Among university students, the prevalence of depression is significantly higher than in the general population. A study conducted by Arevalo Garcia (Arévalo García et al. 2019) found that 38% of Colombian university students have a confirmed diagnosis of depression. According to the World Health Organization (WHO), approximately 5% of adults worldwide suffer from depression, which equates to about 280 million people diagnosed with this disorder. Depression can have serious consequences, including the risk of suicide, a phenomenon that, according to WHO estimates, causes around 700,000 deaths annually (Organización Mundial de la Salud 2023).

A systematic review of 64 studies involving more than 100,000 university students estimated a combined prevalence of depressive symptoms of 33.6% globally (Li et al. 2022). In Colombia, recent studies show equally alarming figures, with prevalence rates ranging from 26.8% to 47.7% in the university population (Restrepo et al. 2022; Londoño et al. 2021). This evidence underscores the need for effective screening and intervention strategies based on valid and culturally appropriate instruments. The impact of the COVID‐19 pandemic exacerbated the prevalence of depressive disorders among young people, prompting PAHO to approve in 2023 the “Strategy to Improve Mental Health and Suicide Prevention in the Americas,” which highlights early detection in educational populations as a priority cost‐effective intervention (PAHO 2023).

The Center for Epidemiological Studies Depression Scale (CES‐D), developed by Radloff 1977, is one of the most widely used tools internationally for assessing depressive symptoms in the general population. Its validity is due to its brevity, psychometric soundness, and high diagnostic accuracy for major depressive disorder in different clinical and community settings (Park and Yu 2021). Although more recent instruments such as the PHQ‐9 or the EPDS have gained popularity (Kroenke 2021), the CES‐D retains relevant advantages for population studies, such as the inclusion of items on positive affect, which allows for a more comprehensive assessment of affective state. In addition, its superior performance in specific contexts (Larsen et al. 2023) highlights the need to carefully select the instrument according to the context and target population.

The CES‐D has demonstrated satisfactory psychometric properties in various countries, including China, Brazil, and Ecuador, and has shown measurement invariance across genders and adequate internal consistency (Niu et al. 2021; Filho and Teixeira 2011; Ramírez et al. 2024; Villalobos‐Galvis and Ortiz‐Delgado 2012). However, its factor structure has been inconsistent across contexts. While the original model proposed four factors (depressive affect, positive affect, somatic symptoms, and interpersonal relationships), multiple studies have reported alternative structures: from two‐factor models to configurations of two, three, or five factors, with variations according to language, age, region, and method of analysis (Niu et al. 2021; James et al. 2020; Shin et al. 2017).

In Colombia, psychometric studies on the CES‐D in the university population are scarce. The most recent was published in 2010 with a sample located in the municipality of Pasto (Villalobos 2010), which limits its generalization and highlights the need to update the validation of the scale in diverse and current educational contexts. Having validated scales in Spanish that maintain solid psychometric properties facilitates comparative epidemiological surveillance between countries and universities, a focus highlighted in PAHO's “A New Agenda for Mental Health in the Americas” initiative, which promotes harmonized indicators to monitor gaps in care. Therefore, the objective of this study is to examine the psychometric properties of the CES‐D in a sample of university students in Bogotá, evaluating its factor structure, internal consistency, and validity as a screening tool for depressive symptoms.

## 2 Method

### 2.1 Study Design

An instrumental study with a cross‐sectional design was conducted to analyze the psychometric properties of the Center for Epidemiological Studies Depression Scale (CES‐D) in the Colombian university population.

### 2.2 Participants

The sample consisted of 1738 university students (792 women and 946 men) from a public university in Bogotá, which has a total of 28,581 students. This sample represents approximately 6% of the total student population of the university. We used quota sampling to ensure proportional representation of different academic subgroups, followed by non‐probability quota‐based sampling with convenience selection within strata, and we included all eligible participants recruited during the fieldwork period; the final sample exceeded the minimum recommended for factor analysis (≥ 10 participants per item) (Kyriazos 2018).

### 2.3 Instrument

The Spanish version of the CES‐D validated by González‐Forteza et al. 2008 was used, with the response scale adapted by Villalobos‐Galvis in a secondary education population in Colombia (Villalobos‐Galvis and Ortiz‐Delgado 2012). The scale consists of 20 items that assess depressive symptoms experienced during the previous two weeks. Response options reflect symptom frequency (in days): 0 = none; 1 = 1–2 days; 2 = 3–4 days; 3 = 5–7 days; and 4 = 8–14 days. Total scores range from 0 to 80. This adaptation meets the time criteria established by the DSM‐IV‐TR manuals, which are maintained in the DSM‐5.

### 2.4 Data Collection Procedures and Techniques

Data were collected digitally via the REDCap (Research Electronic Data Capture) platform, developed by Vanderbilt University, between September 2023 and September 2024. Because recruitment and survey completion took place across multiple points in the academic calendar (e.g., early‐, mid‐, and late‐semester periods), we did not restrict administration to a specific week within the term. REDCap ensures secure, confidential, and standardized data management in accordance with good practices in clinical and epidemiological research (Revelle 2025).

### 2.5 Data Analysis

Statistical analyses were performed in the RStudio environment, using the *psych* (Revelle 2025) and *lavaan* (Rosseel 2012) packages. The complete code used for processing, factor analysis, and reliability estimation is publicly available on GitHub for consultation and replication: https://github.com/agcarod/cesd_analisis.

We assessed univariate normality using the Shapiro–Wilk test. The results indicated significant departures from normality across the CES‐D items (*p* < 0.05), which was expected given the ordinal response format and the typically skewed distribution of depressive symptom indicators in non‐clinical student samples.

An exploratory factor analysis (EFA) was performed using the principal axis method, with Varimax rotation and Kaiser normalization. The number of factors was determined by combining the Kaiser criterion (eigenvalues > 1), the scree plot, and, complementarily, parallel analysis (Horn 1965). For the interpretation of factor loadings, a conventional criterion was applied, considering those equal to or greater than 0.40 as significant (Hair et al. 2013). Correlations between factors were calculated using Pearson's coefficient.

Sample adequacy was assessed using the Kaiser‐Meyer‐Olkin (KMO) index and Bartlett's sphericity test. Given that the items present a five‐level ordinal scale, polychoric correlation matrices and the WLSMV estimator were used, which is appropriate for ordinal data and independent of multivariate normality (Li 2016). Likewise, a second‐order factor analysis was performed using the bifactor model, with the aim of verifying whether the identified factors were related to a higher‐order factor corresponding to depression. Previous studies have supported the use of this model in various scales, showing a better fit than unidimensional models and confirming the presence of a predominant general factor (Baptista et al. 2019; McElroy et al. 2018; Heinrich et al. 2020).

The internal reliability of the scale was estimated using Cronbach's alpha (*α*) and McDonald's omega (*ω*) coefficients, both for the total sample and by gender. Likewise, a hierarchical bifactor model was fitted, from which the following indices were derived: hierarchical omega (*ω*H), explained common variance (ECV), Percent of Uncontaminated Correlations, (PUC), IECV index per item, and ECV_SS (Explained Common Variance—Subscale Specific) and ECV_GS (General‐Specific Explained Common Variance) indices were derived. The latter was used to evaluate the proportion of variance explained by specific factors, once the general factor had been controlled, while ECV_GS allowed us to estimate how much of the common variance in each subscale was attributable to the general factor. The interpretation of these indices followed the criteria proposed by Rodriguez, Reise, and Haviland, considering that values of *ω*H > 0.70 and ECV > 0.60 indicate an essentially unidimensional structure (Rodriguez et al. 2016), while high values of ECV_SS support the independent interpretation of specific factors (Rodriguez et al. 2016).

Finally, measures of central tendency and dispersion were calculated according to the nature of the variables, and cut‐off points differentiated by sex were determined, defined as one standard deviation above the mean (M + 1SD). This criterion was established as an empirical threshold, useful for identifying high levels of depressive symptomatology within the sample distribution, without necessarily implying a clinical diagnostic cutoff point. In addition, to evaluate possible differences between the scores of men and women, an exploratory bivariate analysis was performed using the Mann‐Whitney *U* test, assuming the absence of normality in the distribution of the data. The Bonferroni correction was applied for multiple comparisons in order to adjust the level of significance and control the type I error.

## 3 Results

Regarding sociodemographic characteristics, 53.7% of participants were men, 45.0% were women, and 1.3% identified with another gender identity. The mean age was 21.4 years (SD = 3.4). Most participants belonged to socioeconomic strata 2 (34.0%) and 3 (42.2%), followed by stratum 4 (13.0%), stratum 1 (8.5%), stratum 5 (1.9%), and stratum 6 (0.4%).

### 3.1 Exploratory Factor Analysis (EFA)

First, the indicators of data adequacy for Factor Analysis (FA) were acceptable. On the one hand, the sample adequacy index had a high value (KMO = 0.95), and Bartlett's sphericity test also showed that the correlation matrix between the variables is viable for continuing with FA (*X* ^2^ = 17,902.82, df = 190, *p* < 0.001).

Based on these results, and using Varimax rotation, a configuration of three factors was found, as shown in Table 1. The first factor, called Depressive Affect, explains 33% of the total variance. The second factor, consistent with the Positive Affect dimension, explains an additional 15%, and the third factor, Social Aspects, contributes 8%. Together, the three factors explain 56% of the total variance, suggesting an adequate factor structure for measuring the depressive construct.

**TABLE 1:** Explained variance of the model.

Factor	Eigenvalue	Explained variance (proportion of variance)	Cumulative explained variance
Depressive affect	6.52	0.33	0.33
Positive affect	3.06	0.15	0.48
Social aspects	1.68	0.08	0.56

### 3.2 Composition of Factors

Table 2 shows the grouping of items on the CES‐D scale according to the factor in which they have loadings equal to or greater than 0.40. The Depressive Affect factor concentrates most of the items related to emotional and physical symptoms of depression. The Positive Affect factor includes items with reverse loading that assess positive emotions, while the Social Aspects factor is represented by items that address difficulties in relationships with other people. High factor loadings indicate a strong association between the item and the corresponding factor. The saturation of the items in the factors ranged from 0.46 to 0.84.

**TABLE 2:** Factor loadings of the CES‐D scale items after varimax rotation.

No	Item	Depressive affect	Positive affect	Social aspects	IECV
3	Feeling sad despite support	0.82			0.86
6	Feeling depressed	0.80			0.94
18	Feeling sad	0.79			0.94
7	What you do is an effort	0.68			0.71
20	Not being able to cope with life	0.66			0.99
10	Being afraid	0.63			0.86
5	Having attention problems	0.63			0.62
14	Feeling lonely	0.63			0.99
1	Feeling upset	0.62			0.81
11	Waking up tired	0.61			0.60
2	Having a poor appetite	0.60			0.88
9	Failing in life	0.59			0.99
17	Crying occasionally	0.57			0.86
13	Talking less	0.46			0.95
16	Enjoy life		0.84		0.28
12	Being happy		0.80		0.30
8	Feeling hopeful about the future		0.78		0.25
4	Feeling capable		0.58		0.28
15	Feeling that people are unkind			0.73	0.63
19	Not being liked by others			0.63	0.54

Correlations between the three factors were identified in the scale. There is a moderate correlation between Depressive Affect and Positive Affect (*r* = 0.51), and a stronger correlation between Depressive Affect and Social Aspects (*r* = 0.74). The correlation between Positive Affect and Social Aspects is lower (*r* = 0.35), indicating that, although related, the factors represent distinct dimensions of the depressive construct.

### 3.3 Bifactorial and Hierarchical Factor Analysis

In order to identify whether the three factors found could be combined into a single factor corresponding to the construct of depression, a second‐order factor analysis was used. The indicators obtained with bifactor suggest that the structure of the CES‐D is strongly dominated by a general factor of depression. The hierarchical omega coefficient (*ω*H = 0.85) indicates that more than 85% of the reliable variance in the scores can be attributed to this general factor. Likewise, the common explained variance (ECV = 0.71) reinforces this predominance, since more than 70% of the variance shared between the items is attributable to the general factor. Although the Percent of Uncontaminated Correlations, (PUC = 0.54) is at a moderate level, the high values of *ω*H and ECV allow us to consider that the scale can be treated as essentially unidimensional. These results support the use of a total score as an overall measure of depression in this sample.

The item‐level analysis, based on the Index of Variance Explained by the General Component (IECV), showed that most CES‐D items have high levels of saturation in the general factor. Fourteen of the twenty items showed IECV values above 0.85, indicating that they mainly measure the general depressive construct. However, items related to positive affect (Item 4, Item 8, Item 12, and Item 16) had low IECV values (< 0.31), indicating a weaker relationship with the general factor and a more substantial loading on a specific factor. This differentiation is consistent with the factorial findings and reinforces the idea that the CES‐D is largely unidimensional, although it includes a subset of items that could be interpreted as a distinctive component of positive affect. The same was true for the items that loaded on social aspects in the exploratory FA (Item 19, Item 15), but unlike the previous ones, they had moderate IECV values.

### 3.4 Sample Adequacy and Internal Reliability

Table 3 presents indicators of the quality of the factor analysis and the internal consistency reliability of the scale for the subgroup of men, for the subgroup of women, and for the total sample. In all three groups, the KMO index (≥ 0.94 in all groups) indicates excellent suitability of the sample for factor analysis. Bartlett's sphericity test was significant (*p* < 0.001), which justifies the application of factor analysis for the total sample and the subsamples. The number of factors extracted is the same in all three groups (eigenvalue > 1), the total variance explained is 56% in the complete sample, and the total variance explained in each group was 52%. No differences in factor structure (number of factors and items per factor) were observed between men and women. The scale showed high reliability, with Cronbach's *α* and McDonald's Omega *ω* coefficients above 0.92, with no significant differences between men and women. Likewise, item‐total correlations were adequate, supporting the internal consistency of the items.

**TABLE 3:** PA quality and reliability indicators for men, women, and the total sample.

Indicator	Men (*n* = 946)	Women (*n* = 792)	Total (*n* = 1738)
KMOc index	0.94	0.94	0.95
Bartlett's test			
*χ*2	9195.447	8445.875	17,902.82
Degrees of freedom	190	190	190
*p*	< 0.001	< 0.001	< 0.001
Factors with eigenvalue > 1	3	3	3
Explained variance (%)	52	52	56
Cronbach's *α* coefficient	0.92	0.93	0.93
Omega *ω* coefficient	0.94	0.94	0.94
Item‐total correlation	0.30–0.82	0.45–0.83	0.37–0.82

### 3.5 Analysis by Subfactors

**FIGURE 1:**
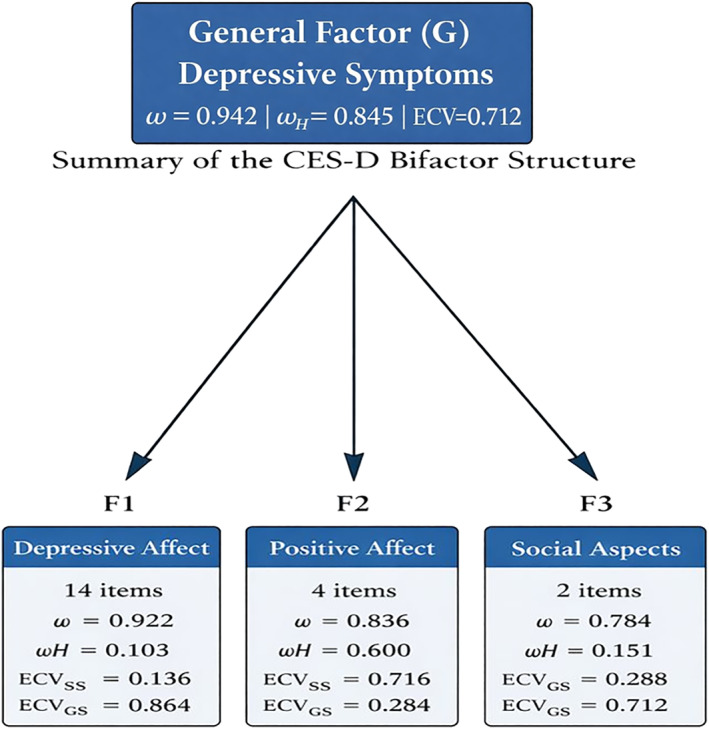
Summary of the bifactor structure of the Center for Epidemiologic Studies Depression Scale (CES‐D) in Colombian university students. The model includes a general factor of depressive symptoms and three specific factors—depressive affect, positive affect, and social aspects. Reliability and dimensionality at the general and factor levels are summarized using total omega (*ω*), hierarchical omega (*ω*H), and explained common variance indices (ECV_SS and ECV_GS).

At the level of specific factors, Figure 1 shows relevant differences in terms of the unique contribution of each dimension. The general factor (G) corresponding to the depression construct showed high reliability (*ω* = 0.94) and a large proportion of explained variance (ECV_SS = 0.71), confirming its dominant role. For its part, the positive affect factor (F2) presented a considerable hierarchical omega (*ω*H = 0.60) and a high specific ECV (ECV_SS = 0.72), suggesting that it represents a significant and distinguishable dimension within the overall construct. In contrast, factors F1 (depressive affect and somatic symptoms) and F3 (social aspects) had considerably low *ω*H (0.10 and 0.15, respectively) and high ECV_GS values (> 0.70), indicating that their variance is mostly shared with the general factor. Taken together, these results indicate that, although the CES‐D can be conceptualized as predominantly unidimensional, the positive affect factor could be reported as an autonomous subscale with clinical and theoretical significance (Figure 1).

### 3.6 Distribution of Scores

**FIGURE 2:**
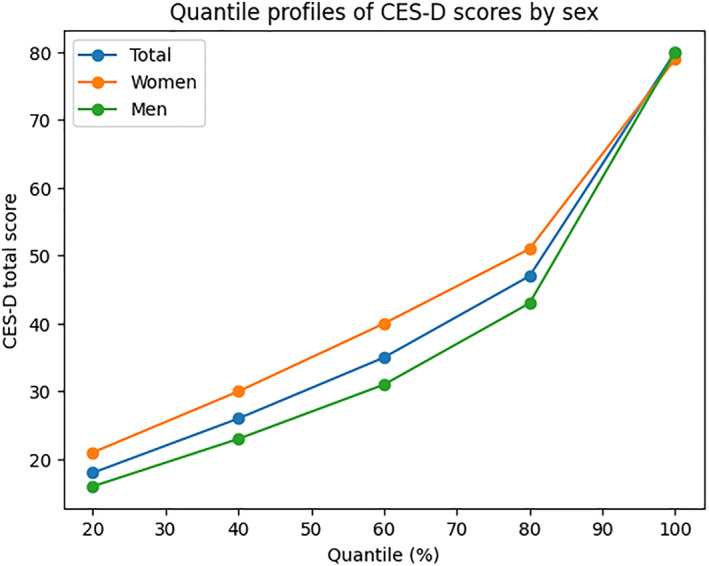
Quantile profiles of total CES‐D scores in the overall sample and stratified by sex. Vertical positions of the curves reflect the empirical distribution of scores across quantiles.

Women obtained significantly higher average scores on depressive symptoms (*M* = 35.89) compared to men (*M* = 29.56), with statistically significant differences according to gender analysis (Mann‐Whitney U, *p* < 0.001). This finding suggests a gender difference in self‐reported depressive symptoms (Figure 2).

Regarding the analysis by socioeconomic strata, no significant differences were observed after applying the Bonferroni adjustment. Although the Kruskal‐Wallis test indicated overall differences between groups (*p* < 0.05), post hoc comparisons did not reveal significant differences between pairs of strata. This could indicate that the difference observed in the overall analysis responds to a general trend in the data, rather than a specific difference between two particular groups. This result may be due to the limited sample size in some strata or to high intra‐stratum variability. Table 4 and Figure 2 present measures of central tendency, dispersion, and distribution by quintiles, allowing for a detailed characterization of the range and distribution of scores in the sample.

**TABLE 4:** Descriptives of the total score among the total sample, men, and women.

Statistic	Total	Women	Men
Mean	32.46	35.89	29.56
Median	30	35	27
Standard deviation	16.01	16.09	15.46
Minimum observed value	0	0	1
Maximum value observed	80	79	80
Quintile			
1	18	21	16
2	26	30	23
3	35	40	31
4	47	51	43
5	80	79	80

Using the criterion of classification corresponding to the mean plus one standard deviation (M + 1SD), it is possible to establish different cut‐off points according to the group analyzed. For the general population, a cut‐off point of 48 is suggested, corresponding to a mean of 32 with a standard deviation of 16. For women, the cutoff point would be 52 (mean = 36; SD = 16), while for men it would be 45 (mean = 30; SD = 15). This approach allows for the identification of high levels of depressive symptoms in relation to the internal variability of each group (Table 4).

## 4 Discussion

The Center for Epidemiological Studies Depression Scale (CES‐D) has been widely used to assess depressive symptoms in university populations internationally, showing diverse factor structures depending on the cultural context and sample characteristics. In the present study, exploratory factor analysis identified a three‐factor solution (depressive affect, positive affect, and social aspects) that explains 56% of the total variance. This structure differs from the original model proposed by Radloff (1977), which proposed four dimensions: depressive affect, positive affect, somatization, and interpersonal relationships.

Variable configurations have been reported in the international literature. In Brazil, for example, Hauck‐Filho and Teixeira (2011) found that the four‐factor model adequately replicated the original structure. In contrast, a multinational study conducted in 27 low‐ and middle‐income countries identified a two‐factor model (depressive affect and positive affect) as the most appropriate (James et al. 2020). In China, (Jiang et al. 2019) reported a three‐factor solution (somatic symptoms, negative affect, and anhedonia) in a shortened 14‐item version. In the Colombian context, Villalobos‐Galvis (Villalobos‐Galvis and Ortiz‐Delgado 2012) found a four‐factor structure in high school students, explaining 55.9% of the total variance. Despite these structural discrepancies, most studies agree that the CES‐D has robust psychometric properties, including high internal consistency, which supports its usefulness as a screening tool in the university population. The variability in factor structure observed between countries and regions reinforces the importance of empirically validating this scale in each specific context before its clinical or research use.

Although exploratory factor analysis revealed a three‐factor structure, hierarchical and bifactorial analyses indicated that the CES‐D behaves as a predominantly unidimensional measure in this sample. The bifactorial model showed a high hierarchical omega coefficient (*ω*H = 0.85) and an explained common variance (ECV) of over 70%, suggesting that most of the variance shared by the items can be attributed to a single general factor of depression. Furthermore, 14 of the twenty items had end IECV indices greater than 0.85, reinforcing the existence of a dominant latent dimension. These findings are in line with previous studies that have applied bifactor models to the CES‐D in university populations. For example, (Fong et al. 2016) found that the bifactor model offered the best empirical representation of the scale structure, highlighting a general factor of depression with minimal specific variance in the secondary factors. Similarly, (Shin et al. 2017) reported high hierarchical omega values for the total score and low values for the subscales, concluding that the use of the overall score is sufficient to assess depressive symptoms. Although other studies have identified different factorial solutions (such as the four‐factor structure replicated in Brazil (Filho and Teixeira 2011) or the modified three‐factor model proposed in China (Jiang et al. 2019)), there is consensus regarding the psychometric robustness of the CES‐D and its validity as an overall measure of depression in university students.

Based on the essentially unidimensional nature of the scale and the observed distribution of scores in the sample, the use of a total score as an overall indicator of depressive symptomatology is proposed, with cut‐off points differentiated by gender: ≥ 52 for women and ≥ 45 for men. These thresholds correspond to one standard deviation above the mean in each group, allowing for the identification of elevated levels of depressive symptoms within the expected variability for this specific population. In line with previous studies (Villalobos‐Galvis and Ortiz‐Delgado 2012; Shin et al. 2017), the findings of this study reinforce the recommendation to use the CES‐D total score as an overall measure of depression, discouraging the isolated interpretation of its subcomponents due to their lower psychometric stability.

The observed unidimensional robustness and gender‐differentiated cut‐off points offer immediate input for the early warning programs that several universities in the Region are implementing in line with the PAHO 2023 regional strategy, as they allow for the identification of students at risk and their timely referral to mental health services (PAHO 2023). Given that depression is a major risk factor for suicidal behavior, the possibility of using the CES‐D as a population screening tool is in line with the regional goal of reducing the suicide rate by 15% by 2030, as set out in both the Sustainable Health Agenda and the PAHO strategy for suicide prevention (Pan American Health Organization 2018; PAHO 2023).

The CES‐D validated in this Colombian population is a starting point for multicenter studies that evaluate university mental health with comparable methodologies in different countries in the Caribbean, Central America, and South America, responding to PAHO's call to generate evidence to guide data‐based regional policies (PAHO/WHO 2025).

Although the findings of this study support the validity and reliability of the CES‐D in Colombian university students, it is important to consider some limitations. First, the cross‐sectional design prevents establishing causal relationships or examining the temporal stability of the factor structure and scores found. Furthermore, although the sample was large, it was restricted to a single public institution in Bogotá, which limits the generalizability of the results to other regions or educational contexts. Second, because data collection spanned September 2023 to September 2024 and took place at different points of the academic calendar, students' symptom reports may have varied with fluctuating academic demands (e.g., end‐of‐term assessments).

Prior longitudinal work suggests that depressive symptoms in university students can peak during high‐demand periods and track perceived academic stress, which could have influenced observed score distributions and, consequently, the proposed cut‐off points (Barker et al. 2018). Although evidence on broader seasonal effects is mixed, future studies should explicitly model timing (semester phase/season) and test measurement invariance and cut‐off performance across periods (Øverland et al. 2019). At the same time, collecting data across diverse academic periods may improve ecological validity by capturing depressive symptoms as they occur in routine university life.

Future research could evaluate the temporal invariance of the factorial model using longitudinal designs, as well as explore criterion validity through comparisons with clinical diagnoses or concurrent measures. Additionally, it would be relevant to examine the sensitivity and specificity of the proposed cut‐off points to optimize their usefulness in population screening contexts. Taken together, these findings reinforce the usefulness of the CES‐D as a valid tool for assessing depressive symptoms in young university students, while underscoring the need for complementary studies that continue to delve into its psychometric behavior in diverse populations.

## Author Contributions

**Angela Gissette Caro‐Delgado:** conceptualization, writing – original draft, methodology, validation, software, formal analysis, project administration, writing – review and editing, data curation, investigation, funding acquisition, resources. **Víctor Rivera Mancilla:** methodology, formal analysis, supervision, writing – review and editing, software, funding acquisition, conceptualization. **Jorge Medina‐Parra:** writing – review and editing, visualization, funding acquisition. **Freddy H. Villalobos‐Galvis:** supervision, writing – review and editing, methodology, investigation, validation.

## Ethics Statement

This study was conducted in accordance with the Declaration of Helsinki. Is part of the doctoral thesis project entitled “Explanatory model of social, interpersonal, and individual factors related to suicidal ideation in students at the National University of Colombia,” which has been endorsed by the Ethics Committee of the Faculty of Human Sciences of the National University of Colombia (Minutes No. 2, March 22, 2023; official letter B.FCH.1.002‐037‐23). A referral protocol to the institution's health services was implemented for students at risk of suicide.

## Conflicts of Interest

The authors declare no conflicts of interest.

## Data Availability

Due to confidentiality and institutional restrictions, the dataset used in this study cannot be made publicly available. However, anonymized data can be obtained from the corresponding author upon reasonable request and after approval by the institutional ethics committee. Additionally, All code used in this study is openly available https://github.com/agcarod/cesd_analisis.
